# Psychological Functions of Semiotic Borders in Sense-Making: Liminality of Narrative Processes

**DOI:** 10.5964/ejop.v13i3.1136

**Published:** 2017-08-31

**Authors:** Raffaele De Luca Picione, Jaan Valsiner

**Affiliations:** a Department of Humanistic Studies, University of Naples “Federico II”, Naples, Italy; b Department of Communication and Psychology, Aalborg University, Aalborg, Denmark; Webster University Geneva, Geneva, Switzerland; University of Neuchâtel, Neuchâtel, Switzerland

**Keywords:** psychological liminality, narrative process, semiotic borders, sensemaking process

## Abstract

In this paper we discuss the semiotic functions of the psychological borders that structure the flow of narrative processes. Each narration is always a contextual, situated and contingent process of sensemaking, made possible by the creation of borders, such as dynamic semiotic devices that are capable of connecting the past and the future, the inside and the outside, and the me with the non-me. Borders enable us to narratively construct one’s own experiences using three inherent processes: contextualization, intersubjective positioning and setting of pertinence. The narrative process – as a subjective articulation of signs in a contingent social context – involves several functions of semiotic borders: separation, differentiation, distinction-making, connection, articulation and relation-enabling. The relevant psychological aspect highlighted here is that a border is a semiotic device which is required for both maintaining stability and inducing transformation at the same time. The peculiar dynamics and the semiotic structure of borders generate a liminal space, which is characterized by instability, by a blurred space-time distinction and by ambiguities in the semantic and syntactic processes of sensemaking. The psychological processes that occur in liminal space are strongly affectively loaded, yet it is exactly the setting and activation of liminality processes that lead to novelty and creativity and enable the creation of new narrative forms.

The human being is continuously engaged in organizing his/her own experiences through the process of narrating (Brockmeier, 1995, 2009; Bruner, 1986, 1990; Polkinghorne, 1998; Sarbin, 1986; Schafer, 1992; Spence, 1982). Narrating one’s own experience always happens “on-line”^i^. This aspect is not always clear and could have deep implications, so at a common sense level it is reasonable to state that narration — talking — is the process of searching for a meaning that occurs *post hoc*, that is, after having lived an experience, we try to find a meaning for it. Yet in a similar vein people can be involved in narrative activities of expected future events even before they happen — narratives of tomorrow’s weather forecast on tonight’s television programs involve the preparation of the subjective apperception for the meaningful nature of the expected future event. Telling stories regarding the expected long-term future outcomes for tellers and their listeners — such as the predictions made by fortune tellers (Aphek & Tobin, 1990), economic experts, or believers in the coming of the “end of the world” (Festinger, Schachter, & Riecken, 1956) involve the narration of expectancies that are at present unknown to the teller and the listeners.

## Borders as Functional Structures

**Figure 1:**
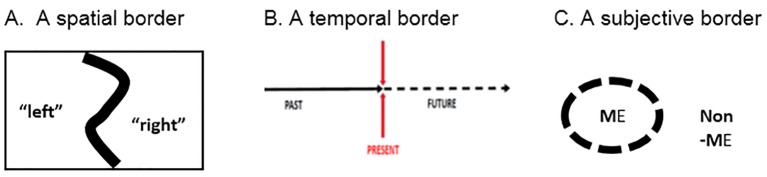
Making distinctions as an act of creating borders.

The aim of this study is to deal with the notion of borders and liminality in order to discuss and broaden the dynamic perspective on the psychological aspects of narrative processes. Liminality implies *temporal*, *spatial, normative* and *subjective* functions of borders (Marsico, 2016; Marsico et al., 2013). Although they are commonly used to demarcate enclosed areas of space, borders can be viewed abstractly as any distinction made within a homogeneous field — be it space, time or distinguishing the Self from the non-Self (“the Other”). Figure 1 illustrates the various forms of borders in their abstract presentation.

Let us consider the crossing of national frontiers, moving from one residence to another, changing one’s job, becoming a parent or suffering from an illness, we immediately feel and perceive our experience in various ways. During all of these different experiences, one’s own contextual semiotic borders are changing. The habitual and consolidated processes for articulating signs in our narrations are no longer available and useful for organizing and guiding our experience, agency and social relations due to the transformation of our semiotic borders. From a referential perspective (based on a static and objective “state of the world” – Bamberg, 2011), we focus on the processes of narration starting from becoming, difference-making, discontinuities and times of crises.

Let us consider two examples of changes in story-telling.

A rupture occurs during an informal meeting between colleagues who are chatting about a workplace episode. The boss suddenly enters the room and the employees stop telling the story. After a brief moment of silence and embarrassment, a situation of uncertainty takes place and after this momentary liminal transition they begin telling a different story about their day. Nothing explicitly prevents them from continuing to tell the previous story, yet the collective recognition of the role of social power played by their boss and the workplace norms establish implicit borders that force the employees to change the topic of conversation.

Another example of borders is triggered by changes in symbolic environments. Let us imagine two friends who are passing through the church doorway at the end of the service. One of them immediately begins telling his friend about his experience as a football player and his team’s hopes of winning the championship. From an attitude of contemplation induced by the sermon that they have just heard, they start discussing trivialities that deeply affect them. The architectural border of the doorway is simultaneously a border between two normative domains for story-telling.

According to Thomas Nail (2016), there are four principal characteristics of borders: borders are in-between, since they are not just the sides of a system that touches another system, borders are a fuzzy zone-like phenomena of inclusive disjunction^ii^ (p. 3); borders are not static but in motion (p. 5); borders are a process of circulation. A perspective on inclusion/exclusion (p. 7) is not enough, «one of the main effects of borders is precisely their capacity to create a hybrid transition zone» (p. 8); the border is not reducible to space (p. 9), the border is a primary process and not a derivative process of spatial ordering.

Crossing borders is a human act (Marsico, 2011; Marsico, Cabell, Valsiner, & Kharlamov, 2013; Valsiner, 2014). Passing from the church to the outside represents a liminal transition^iii^. Every day people pass from home to work, or go to school and back. This is not only physical passing from one location to another — the borders become symbolic thresholds with specified conditions of their passing. A new contextual framework comes into existence together when borders are established, which introduces other discursive possibilities and other kinds of narratives regarding one’s own experiences may unfold as these transitions are taking place. When a person crosses the border from a “traditional” beach to a “nudist” beach at a seaside resort, one tells oneself a story that supports one’s transition through the liminal zone of the new signified part of the seashore. Sign-mediated borders are devices that enable psychological processes of differentiation, opposition, confrontation, connection, categorization and sense-making (De Luca Picione, 2015b; Neuman, 2003; Salvatore, 2016; Valsiner, 2014). We do not use the concept of border in an ontological sense (border as an entity) but from a psychological perspective (borders as semiotic tools that enable dynamic psychic processes – Klempe, 2016).

A border enables us to define our own identity while distancing ourselves from the others in a correlative way at same time. A semiotic border triggers a dynamic process in which the counterpart, the alterity, the otherness, the strangeness are involved. It is impossible to define a Me without a non-Me^iv^. Borders create an essential condition to start a process of identity and narration. A person subjectivises his/her own way of organizing a narration according to the contextual setting of semiotic borders. Borders set the stage for positioning within a symbolic field. Without borders there is no differentiation, no dynamism and therefore no development. Borders have several functions: to create a framework of sense, to diversify subjects and objects and to differentiate identities and positionings. At same time, a simultaneous process of liminality constantly interplays, thus preventing a reification of identity and stiffening of narration regarding one’s own experience. The ongoing construction of semiotic borders and the liminal processes alongside borders are necessary conditions of working. Narrating as an ongoing psychological process of sensemaking implies bordering and liminality.

## From the Breach of Canonicity to the Border Crossing

When a psychological equilibrium is violated, narrative processes are triggered to restore the sense of our experience. This is merely one – and maybe the simplest – of the meanings Bruner discussed in his lesson on narration. Although it is very important, the aspect of breach of canonicity is in fact only one of the wide set of features that make narrative processes so interesting. Bruner defines many other aspects of the process of narration: narrative diachronicity, particularity, intentional state entailment, hermeneutic composability, canonicity and breach, referentiality, normativeness, context sensitivity and negotiability, narrative accrual (Bruner, 1991). In dynamic terms, the narrative process entails a temporal perspective that goes beyond the linear connection between past and present:

I believe that the ways of telling and the ways of conceptualizing […] become recipes for structuring experience itself, for laying down routes into memory, for not only guiding the life narrative up to the present but directing it into the future. (Bruner, 1987, p. 31)

In more general terms, narrating one’s own experience is an active process of construction and connection to the present time of what happened in the past and what it is expected in the future.

## Signs and Borders as Dynamic Devices of Plastic Sensemaking Process

Narration is a process of connection, articulation and integration of fragments of time, experiences and social relations. A person constantly re-configures the narration of his/her own experience contextually and there is a recursive, open, intransitive cycle in mediating self-other-world (Valsiner, 2008). The founding condition of realizing a process of this kind consists in the possibility of using signs (Sebeok & Danesi, 2000). From a semiotic point of view (Lotman, 2005; Neuman, 2003; Salvatore, 2016; Sebeok, 2001; Valsiner, 2007, 2008, 2014), these processes are recursive (Beckstead, 2015) and as such they lead to repeated but always modified efforts to make sense of the current ongoing experience.

In fact, a sign, as well as the emergence, escalation and demolishing of dynamic hierarchies of signs (Valsiner, 2014; Valsiner & De Luca Picione, 2017), provides a meaningful form to experience through acts of differentiation, indication, representation, generalization and reification. It enables us to differentiate between *before* and *after*, between *here* and *there*, between *me* and *non-me* (De Luca Picione & Freda, 2014).

Cancer patients’ narratives provide vivid evidence of such processes. (De Luca Picione, Martino, & Freda, 2017; Martino, De Luca Picione, & Freda, 2016; Martino & Freda, 2016). Patients who had undergone surgery described their experiences and referred to the cutting of the skin and their wounds as a disquieting loss of borders.

“With this cutting on my body, I do not recognize myself, I am no longer able to fend for myself… everything seems so different…”

“I cannot see the wound that surgery has left on my breast, my appearance has changed and I feel confused… I feel as if I lack anchor points, something is broken…”

“…I find it difficult to to narrate my illness experience… I cannot imagine the future, the present is suspended… there is a strain! I feel alone… other people seem so distant…”

In these extracts of narrative texts, serious states of liminality are described. Here the borders of body are affected and the sense of one’s own experience is confused, vague, disquieting (Arcidiacono et al., 2009; Freda et al., 2015; Freda et al., 2016; Rainone et al., 2017; Simão, 2003). The skin and the body are like borders that were somatic, but are now symbolic and social devices at work to differentiate oneself and relate with others - now are unable. The skin has become a semiotic mediating device (Nedergaard, 2016).

Narration implies a process of interweaving and articulation of signs. A plot is an open-dynamic web of signs in which the person lives her own experiences, transcends the present time and learns from and interacts with others. It is an ongoing natural process (Bateson, 1979). Narration means making sense of the integrative capacity of remembering/forgetting the past (Bateson, 1979; Brescó de Luna, 2016; Hoffmeyer, 1996; Neuman, 2003) and anticipating the future (i.e. proleptic imagination) in regard to the changing world and its unpredictability.

Here is the focus point: *narration is always an experience of difference, experience of liminality, experience of creating a space of multiple possibilities*. Narratives often become sclerotic for various reasons and due to a process of naturalization (Barthes, 1964) they are confused with an undisputed and indisputable reality (e.g. the process of acquisition of common sense is based on a consensual taken-for-granted), resulting in "dominant stories" (Castro Cunha et al., 2011; Ribeiro & Gonçalves, 2011) that silence any alternative, thus becoming saturated and unable to transform and evolve. Nevertheless, a narrative is always an ongoing process since the actual action of maintaining, stabilizing or stiffening a story requires energy, effort and actions against transformative pressures arising from comparison, dialogue and differentiation.

Each narrative implies passing through virtual and possible worlds, no-sense places, other points of view, turbulent zones for dismantling and confusing one’s own identity. In our argumentation, we aim to emphasize the semiotic dynamics of signs and their articulation. To this purpose, a deep and extensive discussion of two notions is essential: border and liminality. We intend to discuss various points of view and several theoretical perspectives. Yet, in our opinion, there is an epistemological common ground on which we can deal with the issues of stability/change of making sense of one’s own experience. We intend to take into account the semiotic plasticity of narrative processes, since psychological phenomena are transient and inherently ambivalent. We consider the processes of organization and transformation of sense (De Luca Picione, 2015a; Neuman, 2003; Salvatore, 2016) as dynamic processes that occur along liminal areas created by the organization of borders.

## From Semiosphere to Semiodynamics

The concept of semiosphere was introduced by the Tartu Semiotic School leader Juri Lotman in analogy with the notion of biosphere (Lotman, 2005). It involves the demarcation of the whole domain of human cultural activities in various forms of differentiation of the person and the “outside” of cultural modelling systems. The organization of borders allows for both the stabilization and transformation of the identity process. A border is a device that enables us to distinguish me *versus* non-me (Figure 1C above). It is precisely in this area that processes of sensemaking take place which Lotman regarded as translation processes, aimed at codifying information between one system and another^v^. A simple model of communication involving a sender and a receiver does not cover the complexity of meaningful human relations. A simple tenet of communication is not sufficient (De Luca Picione, Dicé, & Freda, 2015).

In Lotman’s opinion, borders are areas of potentiality, characterized by great instability and faster transformation processes than those at the core^vi^ of the semiotic system. The borders are peripheral spaces in contact with the otherness, the strangeness. The core of the semiotic system is characterized by gradual processes; in a topological sense (De Luca Picione & Freda, 2016a), the central parts of a semiotic system take longer to change and are more stable. Let us consider the normative values in people's lives, the ideologies, the established practices and the habits in attributing meaning that can all be slow in changing.

On the contrary, explosive processes occur along the borders (Lotman, 2009); on the one hand, they are a threat to the stability of the semiotic system, yet on the other hand, they also represent the possibility of development and the integration of new parts^vii^. The borders are able to absorb and to speed up the stimuli of peripheral areas and transmit them to the central structures (De Angelis, 1996). Similarly to a biological membrane (e.g. the membrane of a cell – rich in catalyzing enzymes – predisposes several functions of interaction between the inside and the outside), the border traces a transition profile allowing a dynamic stability between the maintenance of stability and the possibility of transformation.

Therefore we observe that although the notion of the border generally conveys meanings of separation, definition and demarcation between distinct entities, the flip side of the border is constituted precisely by its capacity and function of creating relationships, networking, allowing comparison, exchange and dialogue (De Luca Picione & Freda, 2016b; Freda & De Luca Picione, 2014; Lotman, 2005; Valsiner, 2014). In our opinion the semiosphere notion is limited because it fails to include the temporal side of the inevitable move from past to future in irreversible time (Figure 1B above).

## The Narration Within the Subjective Dynamics of Tension of Borders

Narrative sensemaking of one’s own experience is an ongoing contextual and situated process that originates from the positioning of the narrator with respect to some pertinences (De Luca Picione & Freda, 2014). Contextualization, subjective positioning and pertinentization are processes made possible by creating temporary semiotic borders. Such a dynamicity implies:

1. The definition of a context is not given once and for all but is achieved by organization of wide contextual frames of sense (implicit, unsaid, yet shared and *com*participate) that interpret the present experience and orients through an endless multitude of virtual future possibilities (De Luca Picione & Freda, 2014; Salvatore, 2013; Salvatore & Freda, 2011). Semiotic borders are involved in the organization of discursive and contextual frames.
2. The position assumed by the subject is the effect of his/her relationship with otherness (it is always partly chosen and partly attributed). The subject organizes the cultural significance of his/her own experience through the bordering of mutual positioning with other social actors (Harré & van Langenhove, 1991), who can be both internal and external, both imaginary and real. However, all of the sensemaking processes require a form of otherness against which one can establish a position (Lotman [2005] defines the asymmetry as the basic principle of every dialogical process of sensemaking).
3. Pertinence is the local definition within a wider context of what in a certain moment is relevant and what is not, what is being treated, disputed or negotiated with others. In short, pertinence provides a local form of the object in question and enables a discursive process (De Luca Picione, 2015a, 2015b; De Luca Picione & Freda, 2012, 2014, 2016b; Freda & De Luca Picione, 2013, 2014; Salvatore, 2016).

The configuration of different levels of semiotic borders (consisting of signs and symbolizations) is achieved.

**Figure 2:**
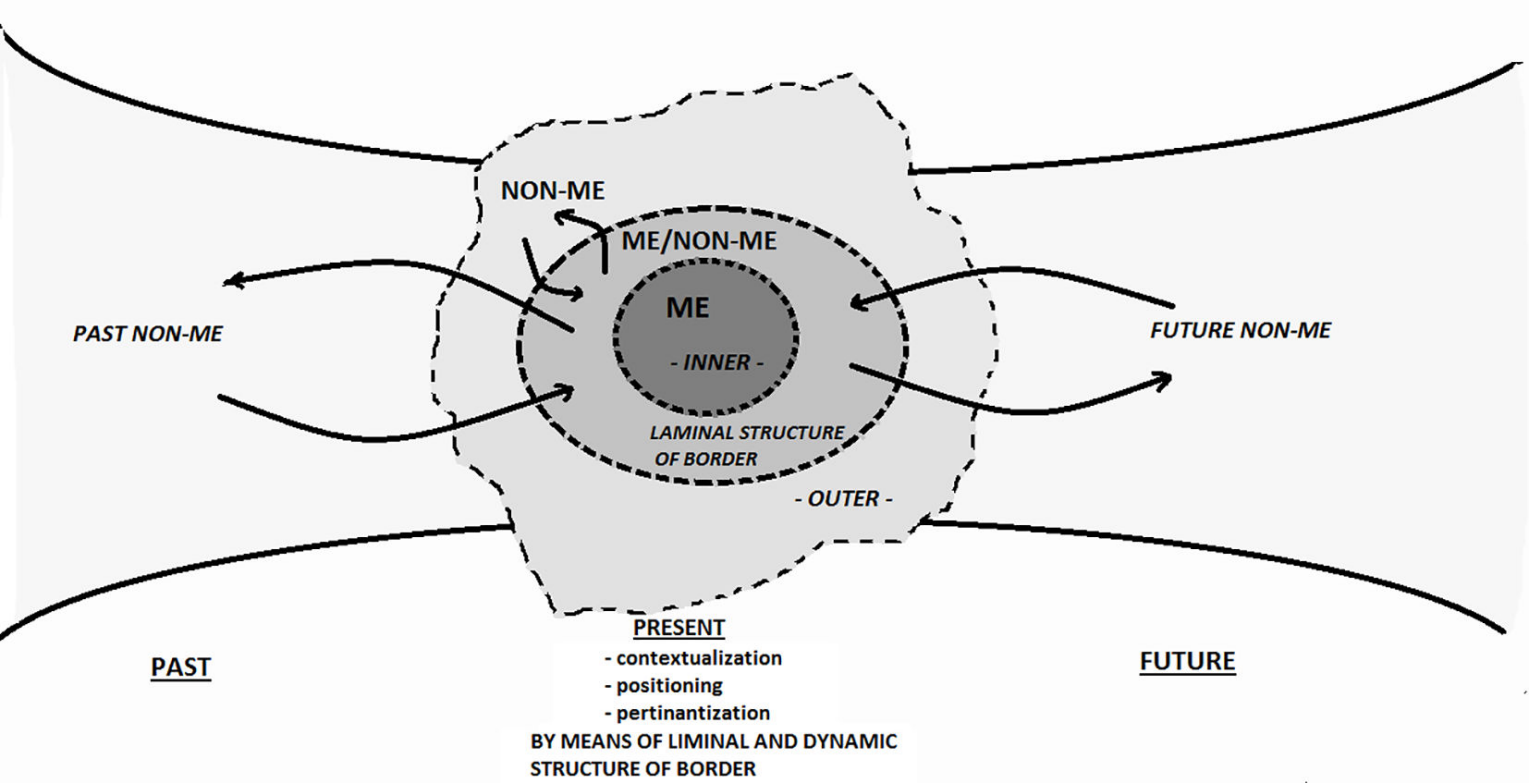
Liminal structure of the border of the self over time.

Rather than drawing a static and unchanging scenario, borders pose a dynamic semiotic grid of reference for actions, relations and narrativization processes. As already mentioned, in psychological terms the borders mainly realize differentiation processes, which are the differences between *before* and *after* (past/future), between *inside* and *outside* (subject/object), between *me* and *non-me* (subject/otherness) (Figure 2).

Figure 2 shows an abstract model of how the Self moves within its immediate context of the non-Self over time. The INNER <> OUTER distinction at each and every moment is “buffered” by the semiotically created layer — the liminal zone of the self. It is the “semiotic skin” that the person superimposes on the biological skin (Nedergaard, 2016) whose function is to allow for the semi-permeable referencing of the past. The ME as it was in the past has become NON-ME over time: when referencing a traumatic event of one’s childhood during adulthood, one’s memories are mediated by the pathways made possible by the current border. Similarly the future — ME-TO-BECOME – is only accessible at present through the pathways afforded by the border of now.

The semiotization by means of borders then allows for the organization of the pillars required for constructing a narrative and initiating a discourse: time, space and the relationship between subjects. These three points are the basis of the deictic process (Bühler, 1934). The borders set up in the present time the incipit to narrate, starting from the differences mentioned above.

Every narration is a process of negotiation and transformation of one’s own relationship with the relational world starting from “borderized” positions where the subject is located. This abstract idea is already rooted in the concept of *mind* according to Gregory Bateson (1979): the mind is the process that unfolds in the relationship between the organism and the environment. Deepening the relevance of differentiation, Greimas (1983) revealed in his narratological studies how the fundamental structure of signification is rooted in a primary process of differentiation. He argued that according to the processes of signification, there is an oppositional process that articulates a semantic micro-universe ruled by a series of fundamental differential relations (Traini, 2013, p. 97). The *semiotic square* of Greimas is a useful tool for studying the processes of sensemaking. It is based on an abstract network of relationships: *opposition*, *contradiction* and *complementarity*. Within this model, the terms are not defined in a substantial manner, but only according to their relationships with one another (Traini, 2013). Not only is the semiotic square useful for analysis and classification but also for creating increasingly complex syntactic sensemaking processes, starting from very basic operations of negation and affirmation. According to Greimas, these transactions outline a path of generativity and designate the embryonal conditions of each narration process^viii^ (Traini, 2013).

## The Transitional Liminal Zone: Possibilities for Stiffening or for Transformation

Generating sense - triggered by processes of differentiation along the border areas – enables us to complexify contextual relations between subject/world/otherness (Simão, 2007, 2013). Simão, when discussing the role of border process in the I/Other/World relationships summarizes this key point very well:

…human living, as they arise from the human potential condition of permanently looking and striving for coherence and stability, on one hand, while unceasingly realizing instability and difference, on the other. This means that what we realize in our I-world relationships we do in terms of pairs of opposites, according to a symbolic imbalanced combinatory movement between and among opposites […] (Simão, 2016, p. 23)

There are two possible directions of development and transformation (De Luca Picione & Freda, 2016b; Freda & De Luca Picione, 2013, 2014). On one hand there is a tendency towards defensive identity processes, stiffening of qualities that become entities^ix^ (Peirce, 1935), hypostatization of the relational system along channeled and normative paths, story of ideological and dogmatic mythology (Barthes, 1964), repetitive and saturating dominant narratives (Castro Cunha et al., 2011; Gonçalves, Matos, & Santos, 2009). On the other hand, we observe tendencies towards the formation of an area of accessibility and multiplicity of possibilities, a virtual place for new connections, a noisy space of polyphony in which the narrative is constructed from various points of view and opinions (Bakhtin, 1981). We observe an open space in which the narration can evolve along various lines of *com*possibilities (many simultaneous "*as if*"-s) and creative mixing of counterfactual conditions (i.e. innovative reformulations of the "*if ..., then ...*") of one’s own experience (Smythe, 2005).

Depending upon the plasticity of borders, there is a continuum from a vision of a rigidly defined reality – from one’s own implicit and social sharing systems of representation and interpretation – towards fertile dynamics of new possibilities and the production of narratives translating the unexpected, the surprise and the uncanny into new messages.

**Figure 3:**
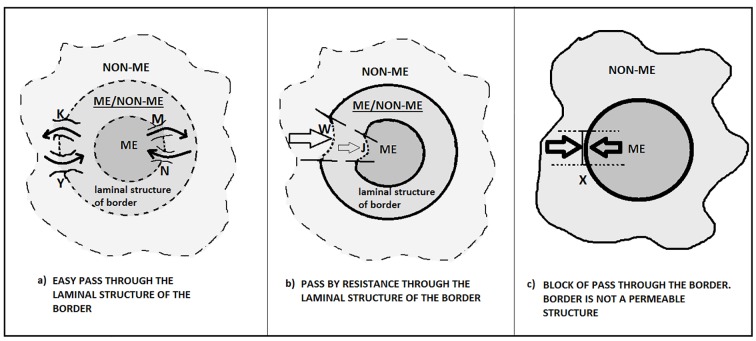
Dynamics of the liminal structure of the border. Under certain conditions (i.e., K, Y, M, N, W, etc. – see the process of catalysis in Valsiner, 2007, 2014), the borders present different dynamics of opening/closure between inner and outer. Three prototypical examples are presented: a. The first case may occur during a workshop in a group setting where the participant encounters other perspectives, opinions, narrations. The opening space of the liminal structure of border - in this friendly atmosphere – enables him/her to create a temporary ambiguous space. In this fuzzy space ME and non-ME are able to create together novel and stories. b. The second case can occur when an insurer tries to convince a potential customer to sign an insurance policy. The former provides various reasons, stories and benefits and encounters the resistance of the latter. If the insurer achieves his/her purpose of persuasion (condition W takes place), he overcomes the resistance (i.e. a border is crossed) of the customer, who starts to tell (condition J takes place) a new story about the advantages of "his choice". c. The third case takes place during a political forum between two candidates from opposing parties. Neither reserves any space of dialogue, so the borders between them are totally non-permeable to the other narrative perspectives. X is just a potential situation of a possible opening but the semiotic system is engaged in a defensive attitude of its core identity. It is blocked thus every possibility of entrance.

The transitional liminal space is the psychological zone in which it is possible to construct new narratives starting from differences (before/after, here/there, me/non-me) (Figure 3). It is essential to cross the liminality for the sake of psychic and social development, although we are somehow aware of the risk and the danger involved. The rituals are symbolic forms aimed at fostering and canalizing a crossing of this kind in order to reach new I-other-world configurations.

## Crossing the Threshold: Intensive Emotionality

In semiotic terms the border crossing is configured as a threshold (De Luca Picione & Freda, 2016c), namely a bifurcation point characterized by the loss of the old symbolic order (meaningful articulations of signs) and by the lack of a future order. The threshold marks the passage by reassuring stability (the previous narrations of self, others and world) to uncertainty and unpredictability (Greco & Stenner, 2017; Salvatore & Venuleo, 2017). According to Lotman (2009), an explosive process is taking place. Such an explosive process marks a discontinuity of unidirectional processes, creating a chaotic, irreversible situation which is potentially open to endless unpredictable possibilities.

A new narration must be imagined and articulated in order to construct a new contextualization, pertinentization and positioning. That will permit a new relational and agentive configuration with the others and the world.

Let us to exemplify this point by revisiting a narrative taken from Heft (2013):

The Inuit of the Nunavut region near Baffin Island rely on hunting and fishing to sustain their way of life, and they routinely travel considerable distances over what is snow and ice-covered terrain for much of the year. During their short summer months, the trails used for this essential travel disappear with the snow melt, and each fall, new trails need to be reestablished. […] How then are the networks of trails long traveled by sled, now with snowmobiles, recreated each year? Aporta (2009) shows that the course of the trails *is woven into narratives of previous trips that are shared between individuals and across generations.* […] Trails rarely are the shortest link between destinations but “usually go through fertile places, across or around lakes, valleys, or open water (on the sea ice) where fish, caribou, and sea mammals can be procured” (p. 10). And it is along the trails where much social interaction occurs, including the sharing of information about fish and game at other points along the trail networks, about travel conditions, and news concerning life events in the dispersed community, such as births, marriages, and deaths. The way-finding information that *is woven into retellings of prior trips seems to be related mostly to features that are to be encountered along the way with little indication that these recountings involve survey representations.* The trails to be followed are presented as itineraries composed of features to be encountered along the way (Heft, 2013, p. 20-21, emphasis added).

It is interesting to note that the potential innovation and the vertigo – with deep and great emotional implications – that liminal areas along borders are able to generate in those who experience this crossing.

## Conclusions: Elaborating the Semiotic Dynamics of Subjective Border Zones

In semiotic terms novelty emerges when the trajectories of routine sense are interrupted and discontinuity is introduced in habitual daily narratives. We are saying that a new narration is possible, in terms of creative exercise, if we overcome the semiotic border that establishes various differences. There is always a reciprocal presupposition between continuity and discontinuity in meaning-making processes (De Luca Picione & Freda, 2014, 2016b; Esposito, Freda, & Bosco, 2015; Freda, 2011; Freda & Esposito, 2017). The *continuity of sense on this side of the border* presupposes the *discontinuity of sense beyond the border* and *vice versa*. In order to be performed narrative processes require an innovative, contingent and contextual configuration that not only marks the breaking of continuity but also a reconfiguration of the old in a new gestalt subject/world/otherness projecting into the future (De Luca Picione, 2015b; De Luca Picione & Freda, 2014, 2016a).

The ongoing creative processes of narration are permitted thanks to the permeability of the borders and the plastic models of life experiences. This perspective leads us to consider the reconfiguration of the borders as social and inter-subjective processes which are also deeply subjective and personal. The plastic and dynamic structure of the border provides the foundation for narrative-based processes of sensemaking of one’s own experience by defining the context, positioning and pertinence. As structure tending towards a dynamic balance, the border is in constant tension between stability and change (De Luca Picione & Freda, 2016a). The psychological implication of this semiotic dynamic is relevant (Tateo, 2013, 2015). In fact, when the border becomes too rigid, we observe forms of repetition of the same narration, a saturation of sense-making processes and a sclerotization of relations based on opposition systems. Similarly, when the border is excessively blurred and fragile, it is impossible to realize a process of signification because it is impossible to define differences and diversity (in certain cases, the discontinuity of experiences could be so extreme that it is impossible to attribute meaning). The experience of liminality – capable of generating new meanings, contributing to the generation of new narrations, projecting the subject into future scenarios – lies precisely in an ongoing, dynamic and contextual tension of oscillatory processes of configuration of semiotic borders.
